# Willingness, concerns, incentives and acceptable remuneration regarding an involvement in teaching undergraduates - a cross-sectional questionnaire survey among German GPs

**DOI:** 10.1186/s12909-018-1445-2

**Published:** 2019-01-25

**Authors:** Tobias Deutsch, Marcus Winter, Stefan Lippmann, Anne-Kathrin Geier, Kristin Braun, Thomas Frese

**Affiliations:** 1 Department of General Practice, Medical Faculty, University of Leipzig, Leipzig, Germany; 2 Institute of General Practice and Family Medicine, Martin-Luther-University Halle-Wittenberg, Halle/Saale, Germany

**Keywords:** Undergraduate medical education, General practice, Curriculum, Teaching, Preceptorship, Preceptor recruitment

## Abstract

**Background:**

Worldwide, many undergraduate general practice curricula include community-based courses at general practitioners’ (GPs’) offices. Usually the academic general practice departments collaborate with networks of affiliated teaching practices. To successfully master the challenge of network development and extension, more information is needed about GPs’ willingness to be involved in different teaching formats, important influencing factors, incentives, barriers, and the need for financial compensation.

**Methods:**

In this cross-sectional study a questionnaire survey was conducted among all GPs working in Leipzig and environs (German postal code area 04). In addition to descriptive statistics, group comparisons and logistic regression were performed to reveal differences between GPs with and without an interest in teaching.

**Results:**

Response rate was 45.3% with 339 analyzable questionnaires. The average age was 52.0 years and 58.4% were women. Sixty-two participants stated that they were already involved in teaching undergraduates. Altogether 60.1% of all GPs and 53.5% among those who didn’t teach yet were basically interested in being involved in undergraduate education. The interested GPs could imagine devoting on average 6.9 h per month to teaching activities. GPs interested in teaching were on average younger, were more actively involved in continuing education and professional associations, and more frequently had pre-existing teaching experiences. The willingness to teach differed substantially among teaching formats. GPs were more willing to teach at their own practices rather than at university venues and they preferred skills-oriented content. Comprehensive organization on the part of the university including long-term scheduling and available teaching materials was rated as most important to increase the attractiveness of teaching. Time restraints and decreased productivity were rated as the most important barriers. Interested GPs appreciated financial compensation, particularly for teaching at university venues, and demanded amounts of money corresponding to German GPs’ hourly income.

**Conclusions:**

The GPs’ interest in undergraduate teaching is generally high indicating a substantial pool of potential preceptors. Recruitment strategies should consider the collaboration with institutions involved in residency and continuing education as well as with professional associations. Comprehensive organization by the responsible department should be promoted and time restraints and decreased productivity should be overtly addressed and financially compensated.

**Electronic supplementary material:**

The online version of this article (10.1186/s12909-018-1445-2) contains supplementary material, which is available to authorized users.

## Background

In Germany as in many other countries worldwide, general practice has become increasingly established as an academic discipline at medical schools during the last decades [1–3]. In many countries the extent of the general practice curriculum within undergraduate medical education has increased accordingly [3–5]. To ensure practice-oriented content and to provide primary care role models inspiring trainees for respective careers, most curricula include courses or clerkships that bring students in touch with GPs working office-based in the universities’ catchment areas [5–8]. For this reason, the German academic general practice departments usually collaborate with networks of GP teaching practices [9, 10]. These networks of specifically trained GPs have to be built up, developed, and maintained [7].

Recent changes in the “Regulation of the Licensing of Doctors” (Approbationsordnung ÄAppO) as well as current plans to reform the German undergraduate medical curriculum called “Masterplan Medizinstudium 2020” strengthen the role of general practice in undergraduate education in adaption to its central importance in health care [11, 12]. Additionally, many medical schools offer extra-curricular teaching formats for students interested in general practice [2]. This leads to a growing demand for GP teachers. For details regarding the basic structure of the German undergraduate medical curriculum please refer to Chenot [13].

To support the recruitment of additional office-based GP teachers, more knowledge about GPs’ willingness to get involved in teaching undergraduates and the factors influencing it is needed [6, 14]. Little is known about differences in GPs’ teaching interest depending on different teaching formats - at university venues as well as at their own practices. Furthermore, it is still necessary to extend the evidence on incentives, barriers, potential “teaching workforce resources” and differences between GPs with and without teaching interest [15, 16]. The present study aimed to disclose the general willingness to teach undergraduates among office-based GPs and how it varies between defined teaching formats. Further objectives were to explore potential associations between teaching interest and socio-demographic and job-related factors, to investigate German GPs’ perceptions regarding incentives and barriers frequently mentioned in the international literature, as well as to reveal the GPs’ perspective on what constitutes adequate financial compensation.

## Methods

### Sampling and design

**Fig. 1:**
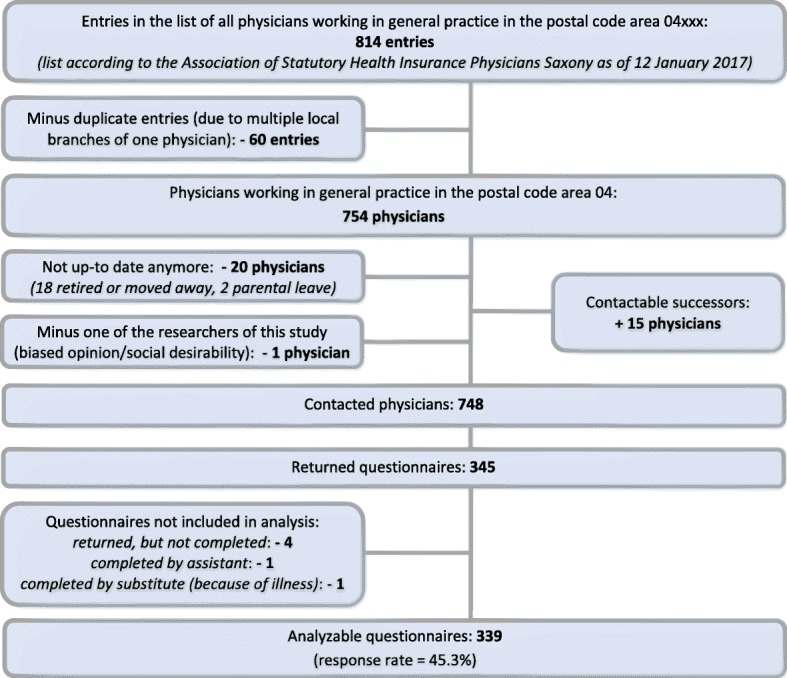
Sampling flowchart

The data of this cross-sectional study are based on a questionnaire survey among all physicians working as general practitioners in the German postal code area 04 according to a list provided by the Association of Statutory Health Insurance Physicians (Kassenärztliche Vereinigung) Saxony as of 12 January 2017. To display the complexity of our research questions the used questionnaire was comparably extensive. Hence, all practices were visited by a colleague and practicing GP (MW) to ensure acceptable response rates (one data collection wave). Visits took place between January 2017 and June 2017. If possible, questionnaires were handed over personally. Questionnaires were accompanied by a formal cover letter explaining the background of the study and the anonymized and statistically aggregated analysis of all data (not allowing personal identification). Participation in the study was voluntary and completion of the questionnaires took place after the visits. Completed questionnaires were sent back by fax or mail. The detailed sampling process can be seen in Fig. 1.

### Non-response-analysis

To allow non-response analysis, all questionnaires were numbered. Response was registered by an independent person in an anonymized list of the contacted GPs. This list only contained questionnaire number, sex, academic degree, specialization, and if the questionnaire could be handed over personally.

### Questionnaire

The questionnaire used in this study was self-developed and contained altogether 86 items addressing the following areas: socio-demographics, professional career, current job-related characteristics, continuing education activities, job satisfaction, interest in teaching undergraduates in general, willingness to be involved in specified teaching formats typically occurring within the German curriculum (including imaginable frequency per year and adequate remuneration), ratings regarding the perceived influence of different incentives on the attractiveness of teaching as well as possible barriers. Questionnaire content regarding potential incentives and barriers was motivated by the literature [16–19]. The final version was the result of a multi-level revision process involving two research-active GPs and two social scientists. Prior to the survey, the questionnaire was pre-tested with a selection of general practitioners representing the target group to ensure general understandability. This led to minor revisions of wording and layout. An English translation of the final questionnaire is presented in Additional file 1.

### Statistics

Data was analyzed using IBM SPSS Statistics 24 for Windows. Considering missing values for single items, frequencies were presented as %_valid_ (n_absolute_/n_valid_). Continuous variables were presented as mean ± standard deviation (SD). For group comparisons regarding frequencies Chi-square test and Fisher’s exact test were used as appropriate. Differences in central tendency were compared with the Mann-Whitney U test. Multivariable binary logistic regression (LR) was performed to reveal variables independently associated with a general interest to be involved in teaching undergraduates. Due to the lack of a pre-existing theoretical model we decided to perform stepwise forward LR model building. Given there was no content-related redundancy or too high correlation with another variable, all relevant variables with univariable group differences on a significance level of *p* <  0.20 as a screening criterion were entered. Statistical significance was assumed for *p* <  0.05.

## Results

### Response and non-response analysis

Altogether 748 physicians were visited and 470 questionnaires (62.8%) handed out personally to the doctors. In the remaining 278 visits (37.2%) it was either handed out to the practice staff or put in the postbox (physician absent due to: visit outside business hours (191), vacation (43), home visits (33), illness (10), continuing education (1)). The response rate was 45.3% with 339 analyzable data sets (Fig. 1).

Physicians who received the questionnaire personally participated more frequently (54.3% (255/470) vs. 30.2% (84/278), *p* <  0.001). There was no significant difference in the participation rate depending on sex (women: 43.9% (198/451), men: 47.5% (141/297), *p* = 0.337). However, we found a higher response rate among those physicians with an academic degree (doctor’s degree or higher: 52.6% (226/430), no title: 35.5% (113/318), *p* <  0.001). In Germany, family medicine is usually delivered by physicians with a specialization in general practice or general internal medicine. A small subgroup has another or no specialization as it was formerly possible to establish a GP practice as a so-called “practical doctor” (Praktischer Arzt) after a certain period of unstructured training [20]. Physicians with a specialization in general practice or internal medicine took part in the study more frequently than those with another or no specialization (general practice: 48.4% (242/500), internal medicine: 41.9% (85/203), others: 26.7% (12/45), *p* = 0.010).

### Description of the sample – Socio-demographics and job-related characteristics

Our sample consisted of 339 GPs. The proportion of women in the sample was 58.4% (198/339) and the participants were on average 52.0 ± 10.3 years old. Further socio-demographic variables as well as job-related characteristics describing the sample are presented in Table 1.

**Table 1:** Socio-demographics and job-related characteristics – total sample and comparison between physicians with and without interest in being involved in teaching undergraduates^*^

Variable	All % (n/n_valid_) ^**^	No teaching interest % (n/n_valid_) ^**^	Interested in teaching % (n/n_valid_) ^**^	p
Female sex	58.4 (198/339)	57.0 (77/135)	59.1 (120/203)	0.705
Age in years (mean ± SD)	52.0 ± 10.3, Median: 52.0	56.2 ± 10.2, Median: 57.0	49.0 ± 9.2, Median: 49.0	**< 0.001**
In a relationship	92.3 (310/336)	93.2 (124/133)	92.1 (186/202)	0.694
Has children	93.7 (312/333)	93.8 (122/130)	93.6 (189/202)	0.918
Has children in the household currently	54.0 (176/326)	41.1 (53/129)	62.8 (123/196)	**< 0.001**
Studied in Leipzig (wholly or at least partially)	82.0 (273/333)	84.7 (111/131)	80.5 (162/201)	0.335
Has a doctor’s degree or habilitation^***^	67.3 (228/339)	66.7 (90/135)	67.5 (137/203)	0.875
Specialist for general practice	71.4 (242/339)	71.8 (97/135)	71.4 (145/203)	0.408
Years since (first) medical specialist exam (whichever) (mean ± SD)	19.2 ± 11.9 Median: 17.0	24.1 ± 12.2, Median: 25.0	15.9 ± 10.3, Median: 14.0	**< 0.001**
Job satisfaction (*scale from 1 = “not satisfied at all* “*to 5 = “very satisfied”)* (mean ± SD)	4.2 ± 0.7, Median: 4.0	4.2 ± 0.8, Median: 4.0	4.3 ± 0.7, Median: 4.0	0.299
Member of the German Society of General Practice and Family Medicine (DEGAM)	8.6 (29/338)	3.0 (4/134)	12.3 (25/203)	**0.003**
Member of the Saxon Society of General Practice and Family Medicine (SGAM)	13.9 (47/338)	6.7 (9/134)	18.7 (38/203)	**0.002**
Member of the Association of General Practitioners (Hausärzteverband)	29.6 (100/338)	26.1 (35/134)	32.0 (65/203)	0.246
Other memberships	26.6 (90/338)	19.4 (26/134)	31.5 (64/203)	**0.014**
No memberships	35.2 (119/338)	44.8 (60/134)	28.6 (58/203)	**0.002**
Has the permission to train residents	37.7 (125/332)	27.7 (36/130)	44.3 (89/201)	**0.002**
A resident is working in the practice	26.3 (86/327)	17.3 (22/127)	32.2 (64/199)	**0.003**
Self-perceived continuing education activities: rather or very high *(*vs. *very low/rather low/average)*	50.7 (170/335)	34.6 (46/133)	61.2 (123/201)	**< 0.001**
Wish to intensify continuing education activities: rather yes/definitely yes *(*versus *definitely not/rather not)*	47.4 (158/333)	36.6 (48/131)	54.2 (109/201)	**0.002**
Years until retirement *(probably)* (mean ± SD)	14.5 ± 8.9, Median: 13.0	10.8 ± 8.1, Median: 9.0	16.8 ± 8.7, Median: 17.0	**< 0.001**
Working in an own practice (versus employed)	79.2 (267/337)	79.3 (107/135)	79.6 (160/201)	0.939
Years having an own practice (mean ± SD) *(refers to those 267 participants with own practices)*	15.8 ± 9.1, Median: 15.0	19.1 ± 8.7, Median: 24.0	13.6 ± 8.8, Median: 12.0	**< 0.001**
Legal structure of the practice:				
Single practice	66.1 (224/339)	71.9 (97/135)	62.6 (127/203)	0.060
Joint practice	24.5 (83/339)	17.8 (24/135)	29.1 (59/203)	
Medical care centre (“MVZ”, including practices of different specialities)	9.4 (32/339)	10.3 (14/135)	8.4 (17/203)	
Practice environment: big city *(*versus *small town/rural area)*	48.9 (160/327)	42.7 (56/131)	53.3 (104/195)	0.061
Average *effective* work time per week (mean ± SD)	46.2 ± 11.3, Median: 46.0	44.8 ± 11.1, Median: 45.0	47.3 ± 11.2, Median: 48.0	0.177
Subjective workload *(scale from 1 =*” *very low* “*to 5 = “very high”)* (mean ± SD)	3.7 ± 0.7	3.6 ± 0.7	3.7 ± 0.8	0.447
Already has experiences with any kind of teaching activity	46.5 (155/333)	19.4 (26/134)	64.4 (128/198)	**< 0.001**

^*^n_valid_ All = n_valid_ no teaching interest + n_valid_ teaching interest + 1 (due to one missing value for the variable teaching interest) ^**^ unless otherwise indicated ^***^ German physicians cannot automatically be addressed as ‘doctor’ after graduation. Thus, it is quite common to engage in scientific activities and to submit and defend a thesis to pursue the academic title ‘Dr. med.’ (≠ PhD). The ‘habilitation’ as the highest postdoctoral degree and prerequisite to apply for a professorial chair is infrequent

### Interest to be involved in undergraduate medical education

Among all study participants, 18.7% (62/332) stated that they were already involved in teaching undergraduates. Including these GPs, 60.1% (203/338) of the entire sample were basically interested in being involved in undergraduate education in the future. Among those who were not teaching yet, 53.5% (144/269) were basically interested doing so. Differences regarding socio-demographic and job-related characteristics between physicians with and without teaching interest are provided in Table 1. The 203 physicians who declared teaching interest could imagine devoting on average 6.9 ± 4.2 h per month to teach medical students (25%-quartile: 4 h, Median: 6 h, 75%-quartile: 10 h).

All participants with a basic interest in teaching students were asked to answer more specific questions addressing their willingness to be involved in defined teaching formats – on campus as well as at their own practices. Besides their general willingness, they were asked to make statements on the imaginable frequency per year and what they would consider to be adequate financial compensation. The respective results are shown in Table 2. On a descriptive level, there was a higher willingness to teach students at their own practices rather than at university venues. At university venues, GPs were more willing to get involved in skills-oriented courses than in examinations or lectures. At their own practices, shorter teaching episodes were more acceptable than longer ones. We found no significant differences regarding the acceptability of an involvement in different teaching formats depending on sex (*p* = 0.105–0.992), except for a higher reluctance on the part of the women to get involved in lectures (‘definitely not’: 62.3% (76/122) vs. 28.2% (24/85); *p* <  0.001) and final examinations (‘definitely not’: 44.2% (53/120) vs. 22.4% (19/85); *p* <  0.001).

**Table 2:** Willingness to be involved in different teaching formats on campus or at own practice (within the group of those physicians who are basically interested in being involved in teaching undergraduates)

**At university venues** …					
Teaching format (with definition as presented in the questionnaire)	Can imagine involvement (rather or definitely yes) *% (n/n_*valid*_*)*	How many times per year? ** *(quartiles)*		Also without remuneration? ** *% (n/n*_*valid*_ *)*	What would be an adequate remuneration? ** *(in Euro, quartiles)*
*Lecture* Tasks: frontal presentation on a defined general practice topic to students in a lecture hall Expenditure of time: 45 min. Lecture + preparation and follow-up + commute; on weekdays	28.6% (56/196)	25%: Median: 75%:	3 4 5	27.5% (14/51)	25%: 60 € Median: 100 € 75%: 150 € *(net per lecture)*
*Skills-training* Tasks: interactive teaching of specific skills needed in general practice (e.g. otoscopy, blood pressure measurement) in small group seminars Expenditure of time: 5 h + commute; on weekdays and possibly on Saturdays	57.1% (112/196)	25%: Median: 75%:	2 4 5	28.6% (28/98)	25%: 100 € Median: 250 € 75%: 400 € *(net per training)*
*Final Examination* Tasks: participation in the oral exam as one out of four examiners and and general practice specialist representative Expenditure of time: approx. 2 × 5 h on two days + preparation + commute; on weekdays	44.6% (86/193)	25%: Median: 75%:	2 2 4	29.9% (23/77)	25%: 200 € Median: 400 € 75%: 600 € *(net for both exam days)*
**At own practice** …					
Teaching format (with definition as presented in the questionnaire)	Can imagine involvement (rather or definitely yes) *% (n/n_*valid*_*)*	How many times per year? ** *(quartiles)*		Also without remuneration? ** *% (n/n*_*valid*_ *)*	What would be an adequate remuneration? ** *(in Euro, quartiles)*
*Elective course general practice (Wahlfach)* Tasks: teaching one student (1st or 2nd study year) at the own practice (e.g. work shadowing and training of basic skills) Expenditure of time: 1 or 2 days, alongside daily work	89.3% (175/196)	25%: Median: 75%:	2 4 10	59.0% (92/156)	25%: 50 € Median: 50 € 75%: 100 € *(net per day)*
*Two-week mandatory general practice clerkship* (Blockpraktikum) Tasks: teaching one student (4th study year) at the own practice Expenditure of time: 30 h within 2 weeks, alongside daily work	83.7% (164/196)	25%: Median: 75%:	2 2 4	45.8% (66/144)	25%: 200 € Median: 300 € 75%: 400 € *(net per clerkship)*
*One-month elective clerkship (Famulatur)* Tasks: teaching one student (3rd to 5th study year) at the own practice Expenditure of time: 4 weeks, alongside daily work	87.4% (166/190)	25%: Median: 75%:	1 2 3	61.4% (89/145)	25%: 350 € Median: 500 € 75%: 600 € *(net per clerkship)*
*Clerkship within the final year (Praktisches Jahr)* Tasks: teaching one student (6th study year) at the own practice Expenditure of time: (currently) 16 weeks, alongside daily work	70.7% (135/191)	25%: Median: 75%:	1 1 2	43.2% (51/118)	25%: 750 € Median: 1500 € 75%: 2000 € *(net per clerkship)*
*Mentorship within a longitudinal general practice curriculum (e.g. LeiKA at University of Leipzig)* Tasks: long-term relationship with a student as a mentor, acting as GP role model, starting at study entry Expenditure of time: per semester: 2 days student at the own practice + 1 day mentor course at university	54.8% (103/188)	–		50.5% (48/95)	25%: 150 € Median: 250 € 75%: 550 € *(net per semester)*

*N = Max. 203 participants with basic teaching interest, Ns vary due to missing values

**N = Max. those participants who could imagine being involved in the respective teaching format

### Attractiveness of and barriers to teaching

Results regarding the physicians’ perceptions of the influence of different incentives on the attractiveness of getting involved in undergraduate education and regarding potential barriers are provided in Table 3 and Table 4. Perfect organization on the part of the university, long-term scheduling and the availability of prepared teaching materials were rated as having the highest potential to increase the attractiveness of teaching, followed by the provision of support to find a practice successor, adequate remuneration, and regular feedback regarding the teaching activities (evaluation). Concerns that teaching is too time-consuming, increases daily work hours and decreases the number of patients treated were the most important barriers.

**Table 3:** GPs’ perceptions regarding the potential of different incentives to increase the attractiveness of teaching undergraduates – total sample and comparison between physicians with and without interest in being involved in teaching undergraduates *(Scale from 0 = “doesn’t affect attractiveness” to + 4 = “very strong increase in attractiveness”)*

Incentive	All (mean ± SD)	No teaching interest (mean ± SD)	Interested in teaching (mean ± SD)	p
Adequate remuneration	1.5 ± 1.3	1.0 ± 1.1	1.8 ± 1.3	**< 0.001**
Special appreciation by Association of Statutory Health Insurance Physicians (Kassenärztliche Vereinigung)/Medical Chamber	1.0 ± 1.2	0.5 ± 0.9	1.3 ± 1.2	**< 0.001**
Official designation as “Academic teaching practice” of the university with certificate	1.3 ± 1.3	0.7 ± 1.0	1.7 ± 1.2	**< 0.001**
Further training addressing *teaching* issues provided by the general practice department at university	1.3 ± 1.1	0.8 ± 1.0	1.6 ± 1.1	**< 0.001**
Further training addressing *medical* issues provided by the general practice department at university	1.4 ± 1.2	1.0 ± 1.1	1.6 ± 1.2	**< 0.001**
Availability of prepared teaching materials	1.8 ± 1.3	1.1 ± 1.2	2.2 ± 1.2	**< 0.001**
Opportunity to participate in shaping the content of the curriculum	1.3 ± 1.2	0.7 ± 1.0	1.6 ± 1.2	**< 0.001**
Perfect organisation on the part of the university	2.0 ± 1.4	1.3 ± 1.3	2.4 ± 1.2	**< 0.001**
Regular feedback regarding the teaching activities (evaluation)	1.5 ± 1.2	0.9 ± 1.0	1.8 ± 1.2	**< 0.001**
Long-term scheduling	2.0 ± 1.3	1.2 ± 1.2	2.5 ± 1.2	**< 0.001**
Access to knowledge (university library, online-books/−journals, etc.)	1.4 ± 1.3	0.9 ± 1.1	1.7 ± 1.3	**< 0.001**
Support to find a practice successor/to recruit medical staff	1.6 ± 1.4	1.2 ± 1.3	1.9 ± 1.5	**< 0.001**
Opportunity to achieve a further academic degree (Dr., habil.)	0.8 ± 1.3	0.3 ± 0.8	1.1 ± 1.4	**< 0.001**
Opportunities for more exchange with other colleagues working office-based	1.5 ± 1.2	1.2 ± 1.1	1.7 ± 1.2	**< 0.001**
Participation in general practice research projects	1.0 ± 1.2	0.6 ± 0.9	1.2 ± 1.2	**< 0.001**

**Table 4:** Potential barriers regarding an involvement in teaching undergraduates – total sample and comparison between physicians with and without interest in being involved in teaching undergraduates *(percentages of participants who ‘rather agree’ or ‘completely agree’ with the presented statements,* versus *‘rather do not agree’ or ‘completely disagree’)*

Statement	All % (n/n_valid_)	No teaching interest % (n/n_valid_)	Interested in teaching % (n/n_valid_)	p
The way to university is too long to be involved in teaching activities there.	33.3 (109/327)	35.7 (45/126)	31.5 (63/200)	0.431
I am afraid that teaching is too time-consuming.	80.1 (265/331)	93.8 (120/128)	71.3 (144/202)	**< 0.001**
I am afraid that the presence of students in my practice will unduly disturb my routines.	38.5 (127/330)	60.2 (77/128)	24.9 (50/201)	**< 0.001**
I fear financial losses caused by teaching.	17.3 (57/330)	20.5 (26/127)	14.9 (30/202)	0.187
I am afraid that supervising students will lead to decreased numbers of patients treated.	54.2 (179/330)	67.7 (86/127)	45.5 (92/202)	**< 0.001**
I am afraid of increased daily work hours caused by teaching.	77.3 (256/331)	85.9 (110/128)	71.8 (145/202)	**0.003**
I do not dare to impart knowledge to others.	11.6 (38/328)	23.4 (30/128)	4.0 (8/199)	**< 0.001**
I am uncertain whether my professional competencies are sufficient for academic teaching.	25.8 (85/329)	41.3 (52/126)	15.8 (32/202)	**< 0.001**
I am uncertain whether my knowledge is sufficiently up-to-date for academic teaching.	30.3 (100/330)	48.4 (62/128)	18.9 (38/201)	**< 0.001**
I feel uncomfortable with the idea that a student is observing me at work.	9.4 (31/330)	19.5 (25/128)	3.0 (6/201)	**< 0.001**
I am afraid that a majority of my patients would refuse the presence of students.	16.4 (54/329)	28.3 (36/127)	9.0 (18/201)	**< 0.001**
I am afraid that the presence of a student would unduly disturb the consultation.	27.1 (89/328)	46.4 (58/125)	15.3 (31/202)	**< 0.001**
I am afraid that the students could pose a risk to my patients.	3.0 (10/329)	3.1 (4/127)	3.0 (6/201)	0.933
There isn’t enough room in my practice to teach students.	27.7 (91/328)	45.2 (57/126)	16.9 (34/201)	**< 0.001**

We found no significant differences between men and women regarding the GPs’ assessments of the potential of various incentives to increase the attractiveness of teaching (*p* = 0.173–0.991). The only exception was a slightly lower interest in possibilities to achieve a further academic degree on the part of the women (0.7 ± 1.2 vs. 1.0 ± 1.4; *p* = 0.048). Regarding potential barriers, women stated significantly more frequently (‘rather agree’ or ‘completely agree’) that they would not dare to impart knowledge to others (14.6% (28/192) vs. 7.4% (10/136); *p* = 0.044), and that they were uncertain whether their professional competencies (30.4% (59/194) vs. 19.3% (26/135); *p* = 0.023) as well as their knowledge (36.6% (71/194) vs. 21.3% (29/136); *p* = 0.003) are sufficient for academic teaching. The assessments of all other potential barriers did not differ depending on sex (*p* = 0.054–0.974).

In our data, there were no statistically significant differences between GPs working in small-town or rural areas and those working in big cities concerning their assessments of the different incentives to increase the attractiveness of teaching undergraduates (*p* = 0.112–0.988). However, with regard to potential barriers GPs from small-town or rural areas agreed (‘rather agree’ or ‘completely agree’) more frequently with the statements that the way to university is too long to be involved in teaching there (50.9% (82/161) vs. 14.8% (23/155); *p* <  0.001), and that they were uncertain whether their professional competencies (31.5% (51/162) vs. 20.5% (32/156); *p* = 0.026) as well as their knowledge (35.6% (58/163) vs. 25.0% (39/156); *p* = 0.040) are sufficient for academic teaching. For all other statements on potential barriers we found no statistically significant differences depending on an urban or non-urban practice location (*p* = 0.080–0.925).

### Variables independently associated with teaching interest

In our sample younger age, memberships in professional associations, higher self-perceived involvement in continued education compared to colleagues, the wish to intensify continuing education as well as existing experiences with any kind of teaching were independently associated with a general interest in teaching undergraduates. Detailed results of the respective logistic regression analyses are shown in Table 5.

**Table 5:** Logistic regression analyses predicting a general interest to be involved in teaching undergraduates (vs. no interest) (2 models: one including and one excluding GPs already teaching undergraduates)

	Model 1 (*including* GPs already teaching undergraduates, *N* = 280) (Nagelkerkes R^2^ = 0.471)		Model 2 (*excluding* GPs already teaching undergraduates, *N* = 225) (Nagelkerkes R^2^ = 0.468)	
Variables included in the model (stepwise forward LR)	Odds Ratio OR (95% CI)	p	Odds Ratio OR (95% CI)	p
Age groups *(*vs. *<= 40 years)*		< 0.001		< 0.001
41 to 50 years	0.57 (0.21–1.53)	0.266	0.67 (0.25–1.82)	0.436
51 to 60 years	0.33 (0.13–0.83)	0.018	0.35 (0.14–0.89)	0.028
> = 60 years	0.04 (0.01–0.15)	< 0.001	0.03 (0.01–0.11)	< 0.001
Any memberships in professional associations *(*vs. *no membership)*	1.97 (1.05–3.70)	0.035	–	–
“rather high” or “very high” self-perceived continuing education activities in comparison to colleagues *(dichotomized,* vs. *“very low”/“rather low”/“average”)*	3.42 (1.79–6.55)	< 0.001	2.82 (1.41–5.65)	0.003
Wish to intensify continuing education activities: “rather yes”/“definitely yes” *(dichotomized,* vs. *“definitely not”/“rather not”)*	1.95 (1.03–3.68)	0.040	2.00 (1.00–3.99)	0.049
Already has experiences with any kind of teaching activity *(*vs. *not)*	10.97 (5.35–22.49)	< 0.001	11.28 (4.85–26.20)	< 0.001

Further variables considered: has children in the household currently *(yes* vs. *no)*, has the permission to train residents *(yes* vs. *no)*, a resident is working in the practice *(yes* vs. *no)*, legal structure of the practice (single practice/joint practice/medical care center), practice environment *(big city* vs. *small town/rural area)*, average effective work time per week *(in hours)*

Variables not considered despite univariable differences: years since (first) medical specialist exam *(reason: correlation with age r = .94)*, years until retirement *(reason: correlation with age r = −.93)*, years having an own practice *(reason: correlation with age r = .81)*

## Discussion

### Summary of the main findings

We found a widespread interest in teaching students, although willingness varied substantially between teaching formats. GPs interested in teaching were generally younger, more actively involved in continuing education and professional associations, and had previous teaching experience. A comprehensive organization on the part of the responsible department including long-term scheduling and available teaching materials was rated as most important to increase the attractiveness of teaching. Time restraints and decreased productivity were the most important barriers. Interested GPs appreciated adequate financial compensation, particularly for teaching at university venues.

### General interest to be involved in undergraduate education

In this study, 60% of all GPs and more than half of those not teaching students to date declared a general interest in being involved in undergraduate education with a median time of 6 hours per month. This interest is promising regarding the necessary extension of the teaching practice networks and is supported by studies from other countries. According to a survey of Gray and Fine, two thirds of UK GPs are interested in teaching over the next 12 months, 44% among those without previous teaching experience [18]. In a study of Baldor et al., US generalists’ interest in future preceptorship was even higher with 92% including those who already taught [17]. On the other hand, Vinson et al. found that 20–30% of US generalists were *actually* teaching students in their practice [15]. Obviously, a basic interest in teaching is not the same as doing it. This is in line with the results of our first subsequent recruitment activities. Some weeks after our survey we contacted all GPs in the same area who had no valid current affiliation contract with our department by mail and offered them to get involved in teaching. This resulted in 28 new affiliation agreements. Compared to more than 50% with a declared teaching interest in the anonymous survey this doesn’t seem to be much. However, it must be considered that we contacted the physicians only once and further public relations work, periodical enquiries and a sound consideration of our insights regarding incentives and barriers may increase the number of new GP teachers.

In our sample, physicians interested in undergraduate teaching were younger and more likely to have children at home, which is in line with the results of other studies [15, 18, 21]. Possible explanations are the greater proximity to the learner’s situation and familiarity with the current medical curriculum, as well as a longer-term professional perspective. It is also conceivable that younger GPs more frequently experienced community-based teaching themselves in medical school, which is known to increase the subsequent likelihood to teach [15]. We found no positive association between the presence of an academic degree and an interest in teaching. A possible explanation might be that the medical doctorate in Germany does not necessarily require academic teaching and that many physicians pursue the degree without an interest in entering university careers. According to our data, physicians interested in teaching are more interested and active in continuing education activities as well as professional societies and associations. This association might be due to a higher commitment towards the specialty of general practice. And it might also be seen in the light of former findings indicating that keeping one’s own knowledge up-to-date is one of the most important rewards of undergraduate teaching as perceived by GPs [17, 18]. We also found GPs with any kind of pre-existing experience with the teacher’s role to be more frequently interested in teaching undergraduates. We cannot clarify with the present study design if this higher interest is caused by a general underlying affinity towards teaching or if a former positive experience as a teacher leads to an increased willingness to teach in the future, or both. However, Morrison et al. reported that US generalist residents expressed greater enthusiasm for teaching, a richer understanding of teaching principles, and a higher willingness to continue teaching 1 year after participating in a randomized trial of a residents-as-teachers curriculum [22]. Consequently, a stronger integration of resident-as-teacher programs into the German GP residency might help to increase the number of doctors with positive teaching experiences and sound teaching skills in the future. In the US resident-as-teacher instructions are widely established as a component of family medicine residency programs [23].

### Willingness to be involved in specific teaching formats and adequate remuneration

We found no data in the literature with which to compare our results regarding the willingness to be involved in specific teaching formats. In summary, it can be said that in our sample the willingness to teach students at the own practice was much higher than the willingness to teach at university venues. Regarding teaching at the own practice, willingness decreased with the length or ‘long-term nature’ of the teaching format. This might reflect a greater caution with regard to long-term commitments. At university venues, GPs were more willing to get involved in skills-oriented courses than in examinations or lectures, which might be an expression of greater uncertainty to impart up-to-date knowledge rather than practical skills. The substantially higher reluctance on the part of the women to get involved in lectures or examinations might be interpreted in the light of their higher uncertainties regarding competencies and knowledge. To counter these gender-specific imbalances, recruitment strategies and further education for new GP teachers should address these obstacles. Regarding financial compensation our results provide insight in the amounts of money a majority of the GP teachers would be satisfied with for different teaching formats. For courses at university venues the necessary time including preparation and follow-up must be considered as a basis of calculation. For courses accompanying daily work at the practice additional time requirements caused by the students’ presence or financial losses caused by decreased productivity, respectively, should be compensated [15]. It is known from former studies that for a majority of GP teachers the presence of a student prolonged daily working time by 30 to 60 min [8, 15, 17]. Considering estimated hourly earnings (median) of 60 to 70 Euros gross (approx. 30 to 35 Euros net) for German GPs [24], the remuneration expectations expressed by the study participants appear to be absolutely appropriate. For teaching formats at university venues GP teachers’ remuneration wishes seem to be based mainly on the expected time requirements. For teaching activities at the own practice their remuneration wishes increase with the length of the teaching format as well. However, this increase is not proportional which may be due to the expectation of the student’s integration in and contribution to everyday practice over time. We should mention, though, that a substantial number of GPs in our sample were willing to teach without remuneration, especially at their own practices. This is in line with other studies showing that GPs motivation to teach is primarily intrinsic [6, 15–17, 19]. On the other hand, incentive compensation for teaching has been reported to be positively associated with motivation in faculty members [25]. Furthermore, it has been shown that for many GPs adequate financial compensation is important and that GP teachers become more uncompromising regarding financial issues over time [18]. Regarding a long-term sustainability of teaching networks an appropriate compensation of the GPs’ efforts seems therefore advisable.

### Incentives and barriers

According to our data, a comprehensive and reliable organization on the part of the responsible department including long-term scheduling and available teaching materials are especially important to enhance the attractiveness of teaching. Results of a recent study from Canada imply that a good support for teaching by the local department is positively associated with the overall job satisfaction of family medicine faculty members teaching students in community-based settings [26]. Other studies described the particular significance of rewards like public recognition, continuing medical education (CME) credits, bookstore discounts, access to computer networks, and workshops to improve clinical teaching [27, 28], which were rated as less relevant in our study. Concerns that teaching will increase working time and decrease productivity were rated as most important barriers. These findings are supported by studies from other countries [18, 19, 28] as well as by a recent survey among German GPs [14]. And indeed, it has been clearly shown that prolonged working time and reduced numbers of patients treated are realistic consequences of teaching students [7, 15, 17, 29, 30]. In our data, non-urban physicians and women stated a higher uncertainty whether their competencies and knowledge are sufficient for academic teaching than their counterparts. This is an interesting finding that might be addressed in further research and should definitely be considered in the recruitment and further education of new GP teachers.

### A brief digression to motivation theories – The wider perspective

Classic motivation theories have suggested that motivation is a context- and time-dependent construct rather than a fixed trait and thus influenceable. Furthermore, a distinction was made between extrinsic motivation, driven by rewards, and intrinsic motivation, which is more desirable as it is driven by personal interest. Autonomy (the person wants to do it), self-perceived competence (capability to achieve the desired goals, expectancy of self-efficacy/ success), and relatedness (to relate or matter to significant others) have been described as important needs that should be satisfied to be intrinsically motivated. A brief overview of some of the classic motivation theories is given by Kusurkar et al. [31]. Our results, particularly the high number of GPs who want to teach and the relatively high percentage of physicians who would teach even without remuneration, imply that there is a high intrinsic motivation to teach among German GPs. The reluctance to get involved in lectures or examinations compared to teaching at the own practice may simply be due to the higher perceived effort and workload. In the context of motivation theory, it could also be seen as an expression of a lower expectancy of success or self-efficacy. Thus, efforts to enhance GPs’ self-perceived competency with regard to teaching in different settings, adequately considering the needs of women and non-urban physicians, seem to be recommendable to increase their intrinsic motivation to teach. Our results regarding the high impact of pre-existing experiences with teaching could be seen in this light as well.

### Strengths and limitations

The results of this study are of importance given its practical implications for the further development of the undergraduate general practice curriculum. Our multi-faceted questionnaire addressed many relevant aspects, including concrete remuneration expectations for defined teaching formats. A sufficient sample size and a comparably good response rate for a complex survey among German GPs support the explanatory power of our findings.

The differences found in the non-response analysis (see methods section) need to be discussed as a limitation. The comparison between responders and non-responders revealed no indication for a sample bias regarding sex. The higher response-rate among physicians who received the questionnaire personally underlines the importance of a peer expressing the request in person. As in this study the (non-)occurrence of personal contact was simply by chance respective systematical bias seems unlikely. However, the significant overrepresentation of physicians with an academic degree (doctor’s degree or higher) as well as with a specialization in general practice or general internal medicine is clearly relevant and should be considered when interpreting some of the findings. Furthermore, we cannot exclude a sample bias by interest in the topic as well as an influence of social desirability. The mentioned limitations may lead to an overestimation of the proportion of physicians interested in teaching. Finally, it should be stated that undergraduate medical education as well as the culture of teaching in medicine partially differ between countries [32, 33]. This might limit the international generalizability of our findings and should be considered when applying our results outside the German context.

## Conclusions and implications for practice

Many German GPs are interested in undergraduate teaching, indicating a substantial and untapped pool of potential GP teachers. As most future GP teachers will be required to provide community-based teaching, the widespread preference to teach at one’s own practice rather than at university is consistent with the needs. Younger GPs who are actively involved in continuing education and professional associations and have previous teaching experiences appear to be most accessible. Recruitment strategies should consider collaborating with the respective institutions and associations. Furthermore, it could be beneficial to implement more opportunities to teach and to acquire teaching skills into the GP residency curriculum. Providing and communicating a reliable and comprehensive organization of teaching by the responsible academic departments may particularly increase the attractiveness of becoming a GP teacher. Time restraints and decreased productivity are the most important barriers and must be overtly addressed. Considering long-term sustainability of teaching practice networks, adequate financial compensation is recommendable. The amounts of money presented in this study appear to be a realistic starting point for resource calculations in Germany. Our results are of interest for GP teachers, general practice departments, medical schools and policy makers.

## Additional file

Additional file 1: English translation of the used questionnaire. The file contains an English translation of the questionnaire used in this study (original language: German). (DOC 162 kb)
