# Supplementary material for: Willingness, concerns, incentives and acceptable remuneration regarding an involvement in teaching undergraduates - a cross-sectional questionnaire survey among German GPs
Source: BMC Med Educ. 2019 Jan 25;19:33. doi: 10.1186/s12909-018-1445-2 (PMC6347773; doi:10.1186/s12909-018-1445-2)
Supplement: Supplementary file 1 — English translation of the used questionnaire. The file contains an English translation of the questionnaire used in this study (original language: German). (DOC 162 kb) [file 12909_2018_1445_MOESM1_ESM.doc]

**Willigness to teach undergraduate students among general practicioners**

Important: Please answer all questions thoroughly. Thank you!

| **Personal details** | | | | | | | | | | | | | | | |
| --- | --- | --- | --- | --- | --- | --- | --- | --- | --- | --- | --- | --- | --- | --- | --- |
| Age: | | |__|__| years | | | | |  | Sex: | | ○ | male | | ○ | female | |
| Are you currently in a relationship?: | | | ○ | yes | ○ | no |  | Do you have childen?: | | ○ | yes | | ○ | no | |
| *If yes*: Number of children currently living in the household: _________________ | | | | | | | | | | | | | | | |
| **Professional career** | | | | | | | | | | | | | | | |
| At which university did you study medicine? ________________________________________________________________________________ | | | | | | | | | | | | | | | |
| Highest academic title: | | | | | ○ | habilitation (postdoctoral  lecture qualification) | | ○ | doctorate | | ○ | diploma | | ○ | none |
| Medical specialty titles: | | | | | | | | | | | | | | | |
|  | Specialist in ______________________________________________________________, since: __________ (year) | | | | | | | | | | | | | | |
|  | Specialist in ______________________________________________________________, since: __________ (year) | | | | | | | | | | | | | | |
|  | Specialist in ______________________________________________________________, since: __________ (year) | | | | | | | | | | | | | | |
| Additional titles/qualifications (please name all and indicate year): | | | | | | | | | | | | | | | |
|  | Additional title ______________________________________________________________, since: __________ (year) | | | | | | | | | | | | | | |
|  | Additional title ______________________________________________________________, since: __________ (year) | | | | | | | | | | | | | | |
|  | Additional title ______________________________________________________________, since: __________ (year) | | | | | | | | | | | | | | |

| Job satisfaction: | | | | | | | | | | not satisfied at all | | not satisfied | | | | partially satisfied | | | | satisfied | very satisfied | | | | |
| --- | --- | --- | --- | --- | --- | --- | --- | --- | --- | --- | --- | --- | --- | --- | --- | --- | --- | --- | --- | --- | --- | --- | --- | --- | --- |
| What is your overall job satisfaction as a general practicioner? | | | | | | | | | | ○ | | ○ | | | | ○ | | | | ○ | ○ | | | | |
| Memberships  (*please name all*): | ○ | DEGAM (German Society of General Practice and Family Medicine) | | ○ | | SGAM (Saxon Society of General Practice and Family Medicine) | ○ | Hausärzteverband (Association of General Practitioners) | | | | | ○ | none | | | | ○ | others: ____________________ | | | | | | |
| Do you have the permission to train residents? | | | ○ | | yes, for specialty in ____________________, since __________ (year), for duration of: ___ months | | | | | | | | | | | | | | | | | | | ○ | no |
| Is a resident currently being trained in your practice?? | | | | | | | | | ○ | | yes | | | | ○ | | no | | | | | | | | |
| Are you currently teaching undergraduates at a university? | | | | ○ | | yes, university of ____________________, since: ___________ (year) | | | | | | | | | | | | | | | | ○ | no | | |

|  | very low | rather low | average | rather high | very high |
| --- | --- | --- | --- | --- | --- |
| How do you perceive your continuing education activities as compared to your colleagues? | ○ | ○ | ○ | ○ | ○ |
|  | definitely not | rather not | rather yes | definitely yes |  |
| Do you wish to intensify your continuing education activities? | ○ | ○ | ○ | ○ |  |

| How many more years will you probably work as a GP before your retirement? | _______________ years |
| --- | --- |
| **Job characteristics** | |

| Type of activity: | ○ | *employed*, for ______hours/week | | | | ○ | working in an *own practice* since:______ (year) | | | | | | | |
| --- | --- | --- | --- | --- | --- | --- | --- | --- | --- | --- | --- | --- | --- | --- |
| Legal structure of the practice: | ○ | single practice | | | ○ | | joint practice | | | | ○ | medical care centre | | |
| How would you describe your practice environment? | | | | | | | | | | | | | | |
|  | | | ○ | big city | ○ | | | small town | ○ | rural area | | |  |  |

| What is your average effective work time per week? | _______________ hours |
| --- | --- |

|  | very low | rather low | average | rather high | very high |
| --- | --- | --- | --- | --- | --- |
| How do you perceive your workload? | ○ | ○ | ○ | ○ | ○ |

| **Interest to teach undergraduate medical students** |
| --- |

| Do you have experiences with any kind of teaching activity? | ○ | yes,: _______________________________________________ (please specify) | ○ | no |
| --- | --- | --- | --- | --- |

| Are you generally interested in teaching undergraduate medical students? | | | | | | | | | |
| --- | --- | --- | --- | --- | --- | --- | --- | --- | --- |
|  |  |  |  | ○ | no | ○ | yes |  |  |

| ***If yes:*** *I can imagine spending ____________ hours per month for teaching undergraduate medical students.* |
| --- |
| ********** If no: please continue directly on page 3 *********** |

| **Willingness to be involved in different teaching formats on campus or at own practice** |
| --- |

| **A: At university venues** | | | | | | | | | | | | | | |
| --- | --- | --- | --- | --- | --- | --- | --- | --- | --- | --- | --- | --- | --- | --- |
| **A1: Lecture**  Tasks: frontal presentation on a defined general practice topic to students in a lecture hall  Expenditure of time: 45 min. lecture + preparation and follow-up + commute; on weekdays | Can you imagine involvement?   definitely not   rather not   rather yes   definitely yes | | How many times per year can you imagine involvement?  _____times per year | | | Could you imagine involvement also without remuneration?   yes   no, only with remuneration | | | In your opinion, what would be an adequate remuneration?  ______________ Euro  *(net per lecture)* | | | | | |
| **A2: Skills-training**  Tasks: interactive teaching of specific skills needed in general practice (e.g. otoscopy, blood pressure measurement) in small group seminars  Expenditure of time: 5 h + commute; on weekdays and possibly on Saturdays | Can you imagine involvement?   definitely not   rather not   rather yes   definitely yes | | How many times per year can you imagine involvement?  _____times per year | | | Could you imagine involvement also without remuneration?   yes   no, only with remuneration | | | In your opinion, what would be an adequate remuneration?  ______________ Euro  *(net per training)* | | | | | |
| **A3: Final Examination**  Tasks: participation in the oral exam as one out of four examiners and and general practice specialist representative  Expenditure of time: approx. 2 x 5h on two days + preparation + commute; on weekdays | Can you imagine involvement?   definitely not   rather not   rather yes   definitely yes | | How many times per year can you imagine involvement?  _____times per year | | | Could you imagine involvement also without remuneration?   yes   no, only with remuneration | | | In your opinion, what would be an adequate remuneration?  ______________ Euro  *(net for both exam days)* | | | | | |
| **B: At own practice** | | | | | | | | | | | | | | |
| **B1: Elective course general practice (Wahlfach)**  Tasks: teaching one student (1^st^ or 2^nd^ study year) at the own practice (e.g. work shadowing and training of basic skills)  Expenditure of time: 1 or 2 days, alongside daily work | Can you imagine involvement?   definitely not   rather not   rather yes   definitely yes | | How many times per year can you imagine involvement?  _____times per year | | | Could you imagine involvement also without remuneration?   yes   no, only with remuneration | | | In your opinion, what would be an adequate remuneration?  ______________ Euro  *(net per day)* | | | | | |
| **B2:** **Two-week mandatory general practice clerkship (Blockpraktikum)**  Tasks: teaching one student (4th study year) at the own practice  Expenditure of time: 30 h within 2 weeks, alongside daily work | Can you imagine involvement?   definitely not   rather not   rather yes   definitely yes | | How many times per year can you imagine involvement?  _____times per year | | | Could you imagine involvement also without remuneration?   yes   no, only with remuneration | | | In your opinion, what would be an adequate remuneration?  ______________ Euro  *(net per clerkship)* | | | | | |
| **B3: One-month elective clerkship** (Famulatur)  Tasks: teaching one student (3^rd^ to 5th study year) at the own practice  Expenditure of time: 4 weeks, alongside daily work | Can you imagine involvement?   definitely not   rather not   rather yes   definitely yes | | How many times per year can you imagine involvement?  _____times per year | | | Could you imagine involvement also without remuneration?   yes   no, only with remuneration | | | In your opinion, what would be an adequate remuneration?  ______________ Euro  *(net per clerkship)* | | | | | |
| **B4: Clerkship within the final year (Praktisches Jahr)**  Tasks: teaching one student (6th study year) at the own practice  Expenditure of time: (currently) 16 weeks, alongside daily work | Can you imagine involvement?   definitely not   rather not   rather yes   definitely yes | | How many times per year can you imagine involvement?  _____times per year | | | Could you imagine involvement also without remuneration?   yes   no, only with remuneration | | | In your opinion, what would be an adequate remuneration?  ______________ Euro  *(net per clerkship)* | | | | | |
| **B5: Mentorship within a longitudinal general practice curriculum (e.g. LeiKA at University of Leipzig)**  Tasks: long-term relationship with a student as a mentor, acting as GP role model, starting at study entry  Expenditure of time: per semester: 2 days student at the own practice +1 day mentor course at university | Can you imagine involvement?   definitely not   rather not   rather yes   definitely yes | |  | | | Could you imagine involvement also without remuneration?   yes   no, only with remuneration | | | In your opinion, what would be an adequate remuneration?  ______________ Euro  *(net per semester)* | | | | | |
| **How would you rate the impact of the following incentives on your interest to teach?** | | | | | | | | | | | | | | |
|  | | ***does not*** *affect attractiveness*  0 | | | ***some*** *increase in attractiveness*  +1 | | ***average*** *increase in attractiveness*  +2 | | | ***strong*** *increase in attractiveness*  +3 | | | ***very strong*** *increase in attractiveness*  +4 | |
| Adequate remuneration | | ○ | | | ○ | | ○ | | | ○ | | | ○ | |
| Special appreciation by Association of Statutory Health Insurance Physicians (Kassenärztliche Vereinigung)/Medical Chamber | | ○ | | | ○ | | ○ | | | ○ | | | ○ | |
| Official designation as “Academic teaching practice” of the university with certificate | | ○ | | | ○ | | ○ | | | ○ | | | ○ | |
| Further training addressing teaching issues provided by the general practice department at university | | ○ | | | ○ | | ○ | | | ○ | | | ○ | |
| Further training addressing medical issues provided by the general practice department at university | | ○ | | | ○ | | ○ | | | ○ | | | ○ | |
| Availability of prepared teaching materials | | ○ | | | ○ | | ○ | | | ○ | | | ○ | |
| Opportunity to participate in shaping the content of the curriculum | | ○ | | | ○ | | ○ | | | ○ | | | ○ | |
| Perfect organisation on the part of the university | | ○ | | | ○ | | ○ | | | ○ | | | ○ | |
| Regular feedback regarding the teaching activities (evaluation)) | | ○ | | | ○ | | ○ | | | ○ | | | ○ | |
| Long-term scheduling | | ○ | | | ○ | | ○ | | | ○ | | | ○ | |
| Access to knowledge (university library, online-books/-journals, etc.) | | ○ | | | ○ | | ○ | | | ○ | | | ○ | |
| Support to find a practice successor/to recruit medical staff | | ○ | | | ○ | | ○ | | | ○ | | | ○ | |
| Opportunity to achieve a further academic degree (Dr., habil.) | | ○ | | | ○ | | ○ | | | ○ | | | ○ | |
| Opportunities for more exchange with other colleagues working office-based | | ○ | | | ○ | | ○ | | | ○ | | | ○ | |
| Participation in general practice research projects | | ○ | | | ○ | | ○ | | | ○ | | | ○ | |
| **Potential barriers regarding an involvement in teaching undergraduates** | | | | | | | | | | | | | | |
| **How do you rate the following statements?** | | | | *completely disagree* | | | | *rather do not agree* | | | *rather agree* | *completely agree* | | |
| The way to university is too long to be involved in teaching activities there | | | | ○ | | | | ○ | | | ○ | ○ | | |
| I am afraid that teaching is too time-consuming. | | | | ○ | | | | ○ | | | ○ | ○ | | |
| I am afraid that the presence of students in my practice will unduly disturb my routines. | | | | ○ | | | | ○ | | | ○ | ○ | | |
| I fear financial losses caused by teaching. | | | | ○ | | | | ○ | | | ○ | ○ | | |
| I am afraid that supervising students will lead to decreased numbers of patients treated. | | | | ○ | | | | ○ | | | ○ | ○ | | |
| I am afraid of increased daily work hours caused by teaching. | | | | ○ | | | | ○ | | | ○ | ○ | | |
| I do not dare to impart knowledge to others. | | | | ○ | | | | ○ | | | ○ | ○ | | |
| I am uncertain whether my professional competencies are sufficient for academic teaching. | | | | ○ | | | | ○ | | | ○ | ○ | | |
| I am uncertain whether my knowledge is sufficiently up-to-date for academic teaching. | | | | ○ | | | | ○ | | | ○ | ○ | | |
| I feel uncomfortable with the idea that a student is observing me at work. | | | | ○ | | | | ○ | | | ○ | ○ | | |
| I am afraid that a majority of my patients would refuse the presence of students. | | | | ○ | | | | ○ | | | ○ | ○ | | |
| I am afraid that the presence of a student would unduly disturb the consultation. | | | | ○ | | | | ○ | | | ○ | ○ | | |
| I am afraid that the students could pose a risk to my patients. | | | | ○ | | | | ○ | | | ○ | ○ | | |
| There isn’t enough room in my practice to teach students. | | | | ○ | | | | ○ | | | ○ | ○ | | |
| **Is there anything you would like to add?** | | | | | | | | | | | | | |  |
|  | | | | | | | | | | | | | |  |

**Thank you for your kind support!**
